# Emotion Regulation Style and Daily Rumination: Potential Mediators between Affect and Both Depression and Anxiety during Adolescence

**DOI:** 10.3390/ijerph17186614

**Published:** 2020-09-11

**Authors:** Neus Zuzama, Aina Fiol-Veny, Josep Roman-Juan, Maria Balle

**Affiliations:** Faculty of Psychology, Universitat de les Illes Balears–Cta. Valldemossa, Km. 7.5-07122 Palma, Spain; a.fiol@uib.cat (A.F.-V.); josep.roman@uib.cat (J.R.-J.); maria.balle-cabot@uib.es (M.B.)

**Keywords:** ecological momentary assessment, adolescents, emotion regulation, positive and negative affect, rumination, mental health

## Abstract

Adolescence is a vulnerable period for depressive and anxious symptom development, and emotion regulation (ER) may be one mechanism linking temperament—i.e., positive affect (PA) and negative affect (NA)—with such symptomatology. Rumination is a common ER strategy that is traditionally assessed using self-reported questionnaires, but it would also be interesting to examine it with an Ecological Momentary Assessment (EMA) approach. Sixty-five adolescents (*M_age_* = 14.69; *SD_age_* = 0.82; range = 14–17 years old; 53.80% girls) completed self-report measures of temperament, ER style, depression and anxiety, and underwent an EMA to investigate rumination use. Results revealed that negative ER style and rumination use mediated the relationship between NA and depression, while only rumination use mediated the relationship between PA and depression. Moreover, NA contributed to increase anxiety, but negative ER style did not significantly mediate this relationship. Rumination use also had no effect on anxiety. This study provides further support for the relationship between temperament, ER, and internalizing problems. It seems that both a negative ER style and rumination use mediate the relationship between NA and depression whereas only NA had a significant direct effect on anxiety. Furthermore, PA buffered the effect of rumination use on depression in this study.

## 1. Introduction

The transition from childhood to adulthood is an especially vulnerable period for the development of psychopathological symptoms and many mental health disorders arise for the first time [1,2,3,4]. During adolescence, depressive and anxiety disorders rank among the most prevalent mental disorders [5], causing a negative impact on adaptive psychological development, including social and academic adjustment [6,7,8].

A high degree of comorbidity between depression and anxiety disorders has been reported several times. As a consequence, several shared etiological mechanisms (transdiagnostic factors) have been proposed [9,10,11,12]. Within these mechanisms, temperamental factors such as high “negative affect” (NA), low “positive affect” (PA), and difficulties in “emotion regulation” (ER) have received increasing support in the development of depressive and/or anxiety disorders (e.g., [13,14,15,16,17]).

Clark and Watson’s tripartite model [18] stands out among the theoretical models searching for the common and specific elements of depressive and anxiety disorders. This model highlights two primary factors: NA (i.e., the tendency to experience negative emotions, such as fear, sadness, and anger) and PA (i.e., the tendency to experience positive emotions, such as enthusiasm and excitement), and a third factor that is physiological hyper-arousal (i.e., the tendency to experience manifestations of tension and somatic activation). According to this model, depression would be defined by a low level of PA and a high degree of NA; whereas anxiety would be defined by high levels of NA and physiological activation. This model has received considerable support in developmental psychopathology for the study of temperamental vulnerability in depressive and anxiety disorders (e.g., [19,20,21,22,23,24,25]). Several studies in this field have found that NA in children and adolescents is associated with general internalizing symptoms (e.g., [13,15,16]). By contrast, PA has been reported to be a protective factor against certain types of psychopathology, mainly predicting fewer internalizing problems during childhood [16]. For instance, PA has been associated with lower levels of depression [13,17]; although it should be noted that some studies with children and adolescents have failed to find PA to be exclusively associated with depression, with these results also suggesting a negative association between PA and anxiety symptoms in community [22,23,25] and clinical samples [19,26]. Nevertheless, it is important to note that many individuals with higher NA and lower PA do not have depressive or anxious symptoms, or do not reach clinical levels. Therefore, one of the research challenges concerns the potential factors that may mediate or moderate the relationship between the temperamental traits of NA and PA and the presence of both clinical or subclinical depressive and anxiety symptoms.

The mechanism most frequently highlighted as a mediator or moderator of the relationship between temperamental factors (e.g., NA and PA) and the presence of depressive and anxiety symptoms is the regulation of emotions. For example, recent studies on this topic have stated that some styles of ER, labeled as poor or negative, mediate the relationship between both NA and PA and depression [27], and between NA and anxiety [28]. In addition, a moderating role of ER has been shown in the relationship between NA and depressive symptomatology [29]. Such studies highlight the importance of ER for a better understanding of psychopathological processes.

Aldao and Nolen-Hoeksema’s [30] widely quoted definition of cognitive ER strategies describes them as “cognitive responses to emotion-eliciting events that consciously or unconsciously attempt to modify the magnitude and/or type of individuals’ emotional experience or the event itself” (p. 974). These cognitive ER strategies are usually divided into positive or adaptive (i.e., associated with beneficial long-term outcomes) and negative or maladaptive (i.e., associated with negative long-term outcomes; e.g., [31,32]). Although the use of either of these strategies depends on context, individuals seem to have a dispositional trend or style to use one type more than the other in many situations [33,34].

Studies that examined cognitive ER style, in both adolescent and adult samples, generally report that poor ER is associated with increased psychopathology, whereas successful emotional coping is associated with lower levels of psychopathology. For instance, several authors [14,35,36,37,38] found that engaging in more negative forms of cognitive ER (e.g., rumination) and, to a lesser extent, not using positive forms to manage negative emotions (e.g., positive reappraisal), predicts a significant number of depressive and anxiety symptoms. Poor cognitive ER style has also been associated with depressive and anxiety symptoms when it manifests together with a high frequency of NA [39].

Among the ER strategies involved in the development of psychological disorders such as depression or anxiety, rumination is perhaps the most widely examined in the literature. Although rumination gives individuals the feeling that they are solving the problem [40] and aims to regulate emotions, it actually triggers a maladaptive outcome [31]. Rumination involves the process of repeatedly thinking on emotional experiences, their causes, and consequences [40]. This suggests that when ruminating, individuals are continually focusing on negative information that is already present in their working memory [41], and have difficulties in updating that information to a more relevant one [42]. Consequently, rumination triggers more negative emotions [43] and fewer positive emotions [44], linking during adolescence with depressive symptoms [45] and anxiety symptoms [46,47].

Different studies have tried to explain the origin of a certain cognitive ER style, considering that variations in temperament probably contribute significantly to emotion management [28,48]. In other words, temperament influences the overall range of emotional responses and preference for ER strategies that children and adolescents initiate to cope with factors perceived as stressful [49,50]. Research in childhood and adolescence indicates that heightened NA is associated with a greater likelihood of dysfunctional ER style engagement [28] and an enhanced use of maladaptive strategies (e.g., rumination; [51]). Conversely, high levels of PA may be protective, since they reduce emotion dysregulation [29] and favor access to more adaptive ER strategies (e.g., cognitive reappraisal; [17]).

At the beginning of the 21st century, ecological momentary assessment (EMA) designs aroused interest in the relationship between rumination and both depressive and anxiety symptoms, and recently interest in this topic has increased; although research has sometimes yielded contradictory results. For example, Silk, Steinberg, and Morris [52] found that adolescents who used rumination more frequently reported more depressive symptoms, while Lennarz, Hollenstein, Lichtwarck-Aschoff, Kuntsche, and Granic [53] concluded that the ER strategies used in response to minor negative events in daily life (including rumination) might not predict depressive symptoms. On the other hand, Tan et al. [54] suggested that rumination may also serve as a risk factor for anxiety, since the effects of rumination were more detrimental to anxious than non-anxious youth.

The role of ER in subclinical symptoms of depression and anxiety in adolescence is not yet well understood. ER has been examined mainly by means of questionnaires and laboratory paradigms, but the information provided is limited. By contrast, the study of ER during adolescents’ day-to-day living has not received as much attention, and EMA may be considered a more well-suited approach to better understand how adolescents regulate emotions on their daily basis than traditionally used cross-sectional designs.

### The Current Study

Given the above, the current study aimed to provide more information on temperament and ER in determining depressive and anxious symptomatology during adolescence in an explanatory study design with latent variables [55]. Since there is evidence of the possibility to predict psychological functioning from the interaction between traits related to ER and a specific emotional response to the situation [50,56,57,58,59,60], we approach ER in two different ways. On the one hand, the individual style of responding to stressful events (ER style, whether negative or positive) and, on the other hand, the use of rumination as a specific ER strategy during adolescents’ daily life.

First, we sought to examine the relationships between adolescents’ temperament (i.e., NA and PA), ER style (both negative and positive), the use of rumination in their daily life, and depressive and anxious symptoms. Having reviewed the relevant literature, we hypothesized that adolescents with high NA would show heightened negative ER style, rumination use during daily life, and depressive and anxious symptoms. In contrast, we expected adolescents with high PA to display greater positive ER style and decreased use of rumination in everyday life and depressive symptoms. No directional hypothesis was made about the association between PA and anxiety symptoms due to the contradictory results provided by the above-mentioned research. In addition, it was expected that a negative ER style would be associated with individuals’ increased involvement in rumination and depressive and anxious symptomatology, whereas a positive ER style would be associated with individuals’ decreased involvement in rumination and depressive and anxious symptomatology. In turn, it was hypothesized that a greater use of rumination during adolescents’ daily life would be related to a greater report of depressive and anxious symptoms.

Second, the literature reviewed in the introduction led us to examine whether adolescents’ temperament (i.e., NA and PA) would be indirectly associated with depressive and anxious symptoms through mediation by their ER style (both negative and positive) and the use of rumination during everyday life. However, since no study to date has investigated both ER style and daily rumination use together as potential mediators of temperament and both depressive and anxious symptoms, the current study aims to address this gap in the literature. We hypothesized that adolescents who report high levels of NA would increase their involvement in a negative cognitive ER style, which would lead individuals to use rumination more often under negative ecological conditions, which in turn would lead to greater depressive symptoms and greater anxiety symptoms; and adolescents who report high levels of PA would increase their involvement in a positive cognitive ER style, which would lead adolescents to use less rumination under negative ecological conditions, which in turn would lead to lower depressive symptoms and lower anxiety symptoms.

## 2. Materials and Methods

### 2.1. Participants

Participants were extracted from a large pool of adolescent students enrolled in a 3-year research project concerning anxiety relationships and ER styles. At the beginning of the project, for the sample recruitment, a total of 15 from 76 high schools of Palma de Mallorca (i.e., 20% of high schools from Palma de Mallorca) were randomly asked to take part in the project. Six high schools rejected to participate, resulting in a total of nine high schools included.

From the nine randomly selected high schools who agreed to participate in the study, the sample initially comprised 72 adolescents. Exclusion criteria were suffering from any diagnosed psychopathological disorder [61] or currently undergoing psychological or psychiatric treatment. As a result, two adolescents were excluded due to the presence of a psychopathological condition (one adolescent presented Major Depression Disorder and another presented Social Anxiety Disorder; *n* = 2 out of *n* = 72, 2.77%). Five participants were also excluded due to a lack of data deriving from technological errors (*n* = 5 out of *n* = 70, 7.14%; see Section 2.4). The final sample was comprised of 65 adolescents (*M_age_* = 14.69; *SD_age_* = 0.82; range = 14–17 years old; 53.80% girls). All participants were Caucasian, from middle-class socioeconomic backgrounds, and from both urban and rural areas. The study was approved by the University’s Research Ethics Committee (5311), and all participants and their parents/legal guardians provided written consent at the beginning of the study.

### 2.2. Materials

#### 2.2.1. Adolescents Psychological Assessment

The Kiddie-Schedule for Affective Disorders and Schizophrenia, Present and Lifetime Version (K-SADS-PL; [62]) is a semi-structured clinical interview designed to identify current and lifetime child psychiatric diagnoses based on the Diagnostic and Statistical Manual of Mental Disorders [61]. We used the Spanish version of the interview, developed by Ulloa et al. [63], which gathers data from both adolescents and their parents. A diagnosis can be coded as definitive, probable (when there is compliance with the diagnostic criteria of 75% or more), or absent. This interview was used to determine whether the adolescents presented any psychiatric disorders. Only participants who were suspected of suffering from a current psychiatric disorder were excluded. The presence of lifetime disorders was also recorded, but was not used as an exclusion criterion.

The Positive and Negative Affect Schedule for Children and Adolescents (PANASN; [64]) is a version of the Positive and Negative Affect Schedule (PANAS; [65]). It was designed to be used with children and adolescents from 7 to 17 years of age, and the original Spanish version was used. This self-report instrument consists of two 10-item scales measuring PA and NA. Participants were asked to rate items according to how they usually feel certain feelings (1 = never, 2 = sometimes, 3 = very often). Cronbach’s *α* for our screened sample was 0.68 for PA and 0.89 for NA.

The Cognitive Emotion Regulation Questionnaire (CERQ; [33]) in its Catalan version [66] was used. This questionnaire consists of 36 self-report items assessing cognitive ER strategies that the individual generally sets up when confronted with a negative or unpleasant event. Items are rated on a 5-point Likert scale ranging from 1 (never/almost never) to 5 (always/almost always). It is composed of nine subscales representing distinct cognitive ER strategies, which may be classified as negative and positive. The negative cognitive ER scales are self-blame, rumination, catastrophizing, and blaming others. The positive cognitive ER scales are acceptance, positive refocusing, refocus planning, positive reappraisal, and putting into perspective. Each subscale score is obtained by summing the individual item scores that correspond to the related subscale (four items each). An overall positive and negative cognitive ER score can also be obtained by finding the total of each subscale score in each category. For this study, we only considered the global positive and negative ER score in the data analysis. The internal consistency in our sample was *α* = 0.91 for the positive scale and *α* = 0.88 for the negative scale.

The Revised Child Anxiety and Depression Scale (RCADS; [67]) in its Spanish version [68] was used. The RCADS is a 47-item self-report questionnaire that assesses anxiety and depressive symptoms thoroughly. The questionnaire covers the following six dimensions of anxiety disorders and depressive disorder: separation anxiety disorder, social phobia disorder, generalized anxiety disorder, panic disorder, obsessive-compulsive disorder, and major depressive disorder. It also provides a scale of total anxiety, which is computed summing the scores of all scales except for major depressive disorder. For the current study, only this total scale and the scale corresponding to major depressive disorder were used. The RCADS requires respondents to rate how often each item applies to them. Items are rated on a 4-point Likert scale (0 = never, 1 = sometimes, 2 = often, 3 = always). The Cronbach’s *α* based on the present data set were 0.94 for the total anxiety scale, and 0.87 for the major depressive disorder scale.

#### 2.2.2. Ecological Momentary Assessment

Using the Clinicovery web tool (https://clinicovery.com) [69], we designed a template for adolescents to report the required data using their smartphones at 24 different time points. Each template consisted of a structured questionnaire, adapted from previous EMA studies on emotional and behavioral functioning (e.g., [54]). The questionnaire was brief, lasting approximately 1 min, and it asked the adolescents about the most negative event they had experienced since last filling in the template, even if it was a minor one.

The adolescents were first asked to indicate whether a negative event had happened or whether everything had been fine since they had last answered the questionnaire, via a dichotomized variable (since the last time I registered the questionnaire, there have been some negative events in which I have experienced negative emotions/since I last registered the questionnaire I have felt good). This item was formulated as follows “Try to remember the emotions and feelings you have had since the last record. Think of the time when you have felt the worst, when you have experienced the most intense negative emotions (e.g., sadness, worry, stress, anxiety, fear, etc.)”.

Where a negative event had occurred, adolescents were asked whether they had used rumination as an ER strategy, via a dichotomized variable (yes/no), to cope with the negative events perceived as stressful. The item was “When you are feeling so bad, have you been constantly thinking about how bad you felt, or the most negative aspects of the problem?”.

### 2.3. Procedure

Participants were accompanied by their parents or legal guardians to the university laboratory. In order to detect current adolescent psychopathological disorders, trained evaluators conducted a semi-structured interview (K-SADS-PL) with each adolescent. Their parents or legal guardians were also given the same psychopathology interview as the adolescent. Afterwards, adolescents completed a battery of self-reported measures (PANAS, CERQ, and RCADS).

A psycho-educational session was then immediately held by the evaluators to inform the participants about rumination strategy. Based on previous research, ER strategy was described in child-friendly, non-technical terms (e.g., [54,70]) to ensure that adolescents would be able to identify the use of rumination when dealing with a negative affective situation, so that they could later report it. During the final section, the app, which was downloaded from Google Play or the Apple Store, was installed onto the adolescents’ cell phones in order to familiarize them with the app’s interface and resolve any doubts.

At home and at pre-scheduled times, the participants were sent a notification through the Clinicovery App©, asking them to complete an electronic template (see the description in Section 2.2.2). At each notification, adolescents were supposed to stop their current activity and complete the template. They were then asked to report whether they had experienced a self-defined negative event that had occurred since their last completion of the electronic template. If they had, the adolescents were to fill in the form. In cases where no event qualified as aversive, adolescents were asked to report whether everything had been okay since they had last answered the questionnaire.

Data were collected over two four-day periods from Friday after school through to the following Monday evening, thus covering an equal number of school and rest days [54]. The adolescents received a notification to complete a template twice between 5 p.m. and 10 p.m. on school days (Friday and Monday) and four times between 11 a.m. and 10 p.m. on Saturday and Sunday, amounting to a total of 24 templates. As noted above, on school days, the adolescents received the notifications in the afternoon to avoid interference with school hours. Each adolescent was given 50 euros compensation for taking part in the study.

### 2.4. Data Acquisition and Pre-Processing

Since we were interested in examining how adolescents regulate their emotions during negative events, we selected only those templates in which individuals reported their occurrence. On average, participants completed 21 out of 24 assessments (87.50%), 39.48% of which (*n* = 539) were situations where the appearance of a stressful event took place, sparking cognitive and behavioral coping processes.

In 27.08% (*n* = 146) of assessments related to ER, adolescents used rumination to cope with the negative events they perceived as stressful. To calculate how often each adolescent had used this ER strategy, the relative use of rumination *ƒ_i_* = *N*_1_/*N* was calculated by aggregating how often rumination was used *N*_1_ = *n*_1_ + *n*_1_ + … + *n*_1_ and dividing this number by the total of ER episodes of each individual *N* = *n*_1_ + *n*_2_ + *n*_3_ +… + *n_k_*.

### 2.5. Analytical Approach

Preliminary descriptive statistics for each variable were performed to determine the appropriateness of mediation models for the data. In addition, differences between males and females in depressive and anxious symptomatology were explored through *t*-tests to rule out the effect of gender on mediation analyses. Likewise, the relationship between age and depressive and anxious symptoms was evaluated using Pearson’s bivariate correlations analysis to rule out the effect of age on mediation analyses. Correlation analyses were also performed to analyze associations between all variables of interest: NA, PA, negative ER style, positive ER style, the relative use of rumination during the EMA, and both depressive and anxiety symptoms.

**Figure 1:**
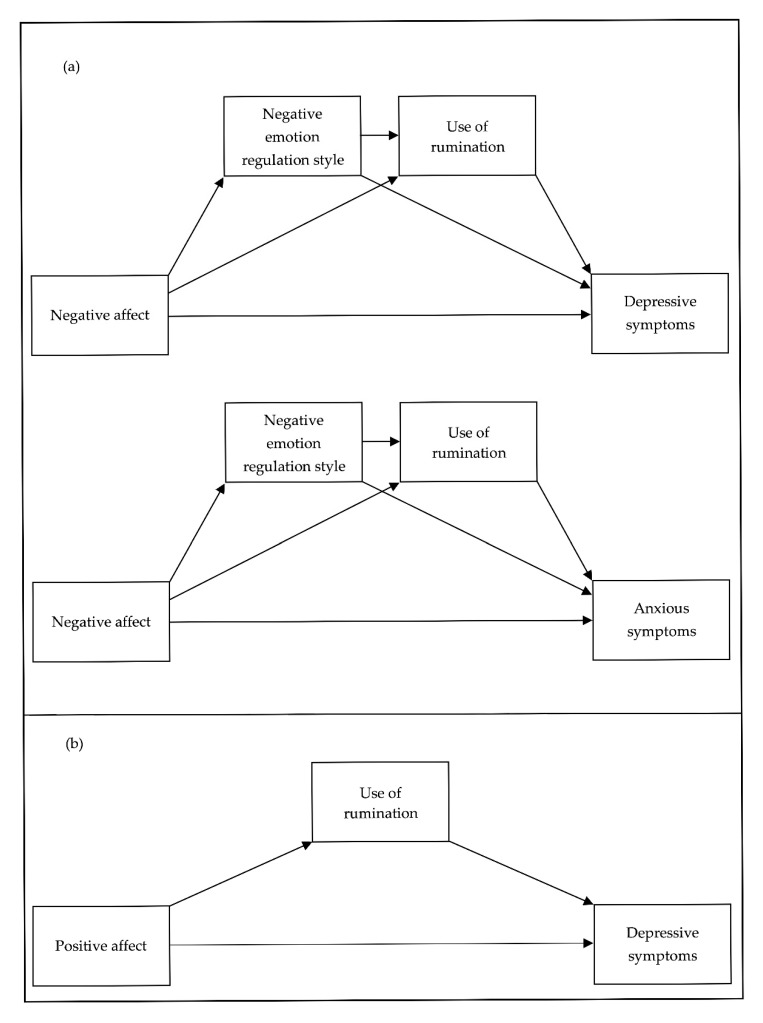
(**a**) The general outline for the multiple mediation models; (**b**) the general outline for the simple mediation model.

Subsequently, three overall theoretical models relating temperament (i.e., NA and PA), ER style (negative and positive), daily rumination use, and depressive and anxious symptomatology were evaluated using bootstrap mediation analyses (specifically two multiple mediation models and one single mediation model). The models were established based on the results obtained in the correlations. Subsequently, we proceeded to fit the model to the data when there were significant associations between the hypothesized predictors, mediators, and outcome, which satisfied initial steps for establishing mediation (as per [71]). Since PA did not show a significant correlation with anxiety symptoms (outcome variable), as explained later in Section 3, the corresponding mediation model was not carried out. Regarding multiple mediation models, the first one was designed to test indirect effects of NA, negative ER style scores, and the use of rumination during the EMA on depressive symptoms (Model 1). The second one aimed to test indirect effects of NA, negative ER style scores, and the use of rumination during the EMA on anxiety symptoms (Model 2). In these models, NA was entered as a predictor, and both negative ER style and the use of rumination were entered as mediators in order to assess their effect on depressive symptoms (in Model 1) and anxiety symptoms (in Model 2). On the other hand, since positive ER style did not show significant correlations with the use of rumination (see Section 3), it was not included as a mediation variable in the corresponding multiple mediation model. Therefore, a bootstrap simple mediation analysis was performed to test indirect effects of PA and the use of rumination during the EMA on depressive symptoms (Model 3) in which PA was entered as a predictor and the use of rumination was entered as a mediator to assess their effect on depressive symptoms. The general outlines used for the different multiple and simple mediation models are presented in Figure 1.

Multiple and simple mediation analyses were conducted using the macro PROCESS version 3.4 in SPSS (SPSS Inc., Chicago, IL, USA) [72]. The number of bootstraps was set to 10,000 with confidence intervals (CI) of 95%. Additionally, due to high statistical correlation between both outcome variables, depression was entered as a covariable into the models testing anxiety, and anxiety was a covariable in those for depression. We also included as covariables the study variables that were statistically correlated with the outcome variable but were of no interest in the corresponding mediation analysis [73]. Specifically, NA and anxiety symptoms were included as covariables in Model 1; positive ER style and depressive symptoms in Model 2; NA, negative ER style, and anxiety symptoms in Model 3.

All analyses were performed using version 25 of the IBM SPSS Statistics software package. A significance level of *p* ≤ 0.05 was adopted.

## 3. Results

### 3.1. Preliminary Analyses

In the preliminary descriptive statistics for each variable included in the different mediation models, the stem-and-leaf plots indicated that the data were not distributed normally (see Table 1). However, the skewness of the distributions ranged from −0.149 to 1.660 and the kurtosis ranged from −0.601 to 3.430. Slight skewness and kurtosis deviations from zero indicate little or no departure from normality, justifying the use of mediation models on our data. Moreover, further visual inspection of the data did not reveal outliers. Regarding anxiety and depression symptoms, skewness and kurtosis values indicate non-Gaussian distributions. This should not be surprising since data were obtained from a non-clinical sample.

Preliminary analyses were also conducted to test for relations between demographic variables and depressive and anxious symptoms. Results showed that depressive and anxiety scores did not differ by gender (Depression: *t*(63) = −1.288, *p* = 0.203; Anxiety: *t*(63) = −1.022, *p* = 0.311) and were not correlated with age (Depression: *r* = −0.157, *p* = 0.213; Anxiety: *r* = −0.116, *p* = 0.359).

### 3.2. Descriptives and Bivariate Correlations

Table 2 shows the pairwise zero-order correlations between adolescent temperament (i.e., NA and PA), ER style (both negative and positive), the use of rumination, and depressive and anxiety symptomatology. Overall, there were significant associations between most of the variables, and most relationships were found to be in the hypothesized directions. Associations between NA and both depressive and anxious symptoms were high and positive, whereas the association between PA and depressive symptoms was low and negative, and no significant correlation was found between PA and anxiety symptoms (*p* > 0.050). Both associations between negative ER style and depressive and anxiety symptoms were positive and high. No significant association was found between positive ER style and depressive symptoms (*p* > 0.050), while the relationship between positive ER style and anxiety symptoms was low and positive. There were positive associations between the use of rumination and both depressive and anxiety symptoms, moderate and low, respectively.

### 3.3. Mediation Analysis

As described in Section 2 above, since PA only correlated with depressive symptoms, the corresponding mediation analysis was not performed with anxiety symptoms. Furthermore, since positive ER style showed no significant correlation with the use of rumination, it was not included as a mediating variable in the last model (i.e., model with PA as a predictor variable of depressive symptoms, through the use of rumination during daily life), so in this case the mediation model was simple.

**Figure 2:**
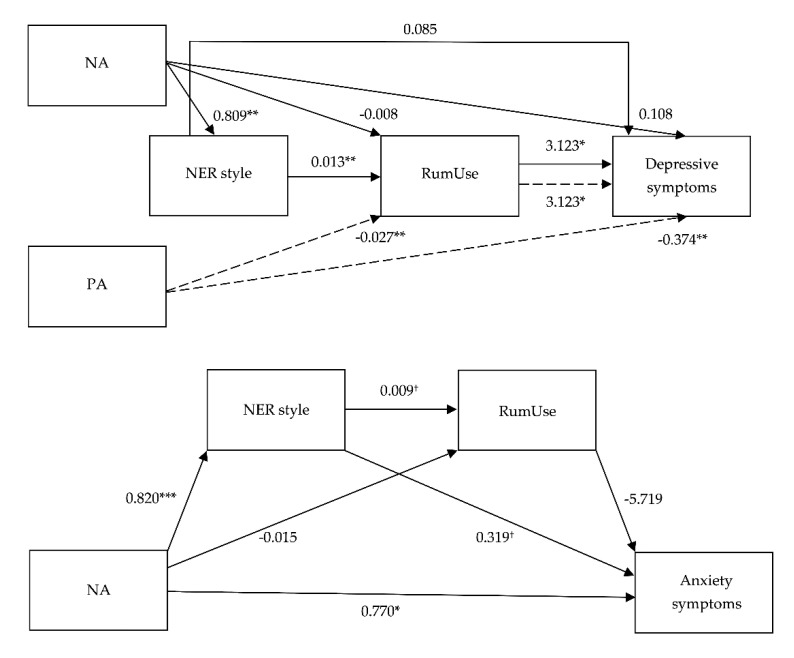
Path diagram depicting all direct paths in the mediation models, with negative affect (NA) and positive affect (PA) as predictors, negative emotion regulation (NER) style and the use of rumination (RumUse) specified as mediators, and depressive symptoms (upper) and anxiety symptoms (lower) as the outcome. All numbers indicate unstandardized path coefficients. ^†^ indicates *p* < 0.10, * indicates *p* < 0.05, ** indicates *p* < 0.01, *** indicates *p* < 0.001.

Coefficients for the regression analyses are presented in Table 3 and Figure 2. Confidence intervals of the indirect effects are presented in Table 4.

Regarding Model 1, although no direct effect was observed of NA on depressive symptoms, the model revealed a direct effect of NA on negative ER style, and from this negative ER style on the use of rumination, but only the use of rumination had a significant direct effect on depressive symptoms (*F*(5, 59) = 43.991, *p* < 0.001, *η*^2^_p_ = 0.788). Thereby, the relationship between NA and depressive symptoms was significantly mediated by both negative ER style and the use of rumination *B* = 0.033, *SE* = 0.023 (95% bootstrapped CI: 0.002, 0.089). Specifically, NA through the mediating effect of negative ER style and the use of rumination, explained 78.85% of the variance in depressive symptoms. It is worth mentioning that part of this explained variance is due to the influence of anxiety symptoms and PA, i.e., covariables in this mediation model, since both variables showed direct effects on depressive symptomatology (Anxiety: *B* = 0.175 (95% bootstrapped CI: 0.111, 0.239), *t* = 5.505, *p* = 0.000; PA: *B* = −0.374 (95% bootstrapped CI: −0.593, −0.155), *t* = −3.423, *p* = 0.001).

As for Model 2, there was no significant mediating effect of NA on anxiety symptoms through negative ER style and the use of rumination during daily life sequentially. In this case, the model only showed a direct effect of NA on anxiety symptoms (*F*(5, 59) = 34.638, *p* < 0.001, *η*^2^_p_ = 0.745), indicating that adolescents with high NA scores reported more symptoms of anxiety. However, despite being a non-statistically significant value, negative ER style tended to have some degree of direct effect on anxiety symptoms, and this negative ER style had an approximately trending indirect effect on the relationship between NA and anxiety symptoms. Specifically, the general model accounted for 74.59% of variance in anxiety symptoms. It should be noted that depressive symptoms were also introduced into the general model as a covariable and had a significant direct effect: *B* = 1.710 (95% bootstrapped CI: 1.009, 2.411), *t* = 4.882, *p* = 0.000 on anxiety symptomatology. In contrast, the other covariable in this model, i.e., positive ER style, showed no direct effect on anxiety symptoms.

With regard to Model 3, both PA and the use of rumination had significant direct effects on depressive symptoms (*F*(4, 60) = 49.545, *p* < 0.001, *η*^2^_p_ = 0.767). Indeed, the relationship between PA and depressive symptoms was mediated by the use of rumination *B* = −0.086, *SE* = 0.047 (95% bootstrapped CI: −0.192, 0.009), indicating that adolescents who report high levels of PA used less rumination under ecological-negative conditions, which in turn prompted them to notify lower depressive symptoms. Concretely, the general model accounted for 76.76% of variance in depressive symptoms. It should be noted that anxiety symptoms were also introduced into the general model as a covariable and had a significant direct effect *B* = 0.175 (95% bootstrapped CI: 0.111, 0.239), *t* = 5.505, *p* < 0.001 on depressive symptoms. In contrast, the other covariables in this model, i.e., NA and negative ER style, showed no direct effects on depressive symptoms.

## 4. Discussion

Depressive and anxiety symptoms are among the most prevalent psychopathological issues in adolescence [5], thus the study of factors involved in their etiology (e.g., temperament and regulation of emotions) is especially important. More research is needed on the relationships of temperament and ER with subclinical symptoms of mental disorders in adolescence; therefore, this article aims to provide more information on both factors in determining depressive and anxious symptomatology during this period of development.

An important strong point of the current study is its ecological validity. The rumination use was evaluated in a naturalistic context, which minimizes the problems associated with retrospective recall as it facilitates the recording of the rumination use just at the moment when the adolescent has experienced negative emotions facing negative daily events. In addition to the ecological validity of the data, the two four-day sampling periods provided more comprehensive and generalizable data of rumination use in adolescents’ daily lives than single-set point assessments, thus discerning the pattern of this ER strategy for each adolescent.

The first aim of this study was to examine the relationships between adolescents’ temperament, cognitive ER style, rumination use in their daily life, and depressive and anxious symptoms. The findings relating temperament and depressive and anxious symptoms revealed, as expected, a significant positive relationship between NA and both depressive and anxious symptoms, while PA was negatively related to depressive symptoms. These results are in line with Clark and Watson’s tripartite model [18] and with the abundant research that has identified NA as a significant risk factor for depression and anxiety in adolescence, whereas low PA has only been associated with depressive symptoms (e.g., [13,22,74]). The hypothesis that cognitive ER style would be related to symptoms of depression and anxiety was partially supported. Our findings revealed that a negative cognitive ER style was positively associated with symptoms of depression and anxiety, in line with research identifying a maladaptive cognitive coping style as a risk factor for depression and anxiety in adolescents (e.g., [14,35]). However, contrary to our hypothesis, no association between positive ER style and depressive symptoms was found, perhaps because there are other factors (e.g., family, cultural, and personal) involved in this relationship. For example, as indicated by Yildiz and Kizildağ [27], an adolescent with certain family and social conditions may exhibit depressive symptoms, even though he or she is employing positive ER strategies to cope with feelings about academic failure. Surprisingly, also against our expectations, a low positive association between positive ER style and anxiety symptoms was found. A recent study with adolescents also failed to find an inverse relationship between positive ER styles and anxiety symptoms [75]. It is worth mentioning that only the overall positive ER score was used for this study (i.e., the sum of all subscales: acceptance, positive refocusing, refocus planning, cognitive reappraisal, and putting into perspective), since in a previous study even though cognitive reappraisal related to less notification of anxious symptoms, other positive coping strategies such as acceptance and positive refocusing showed positive relationships with anxiety symptoms in adults [35]. Perhaps for this reason the expected negative relationship disappears and even becomes positive.

Rumination has been studied mainly through questionnaires that assess its habitual use, but the present study sought to go further and examine the use of rumination during adolescents’ everyday life. Consistent with previous studies, adolescents in this study who most frequently used rumination daily reported more depressive [45,52] and anxiety symptoms [46,47]. This study also examined the relationship between cognitive ER style (i.e., ER as a “trait”) and rumination use during daily life (i.e., ER as “state”). Given the above-mentioned research that indicates the existence of a dispositional trend or style to use one type of ER strategy more than another in many situations, it can be expected that individuals with a negative ER style use rumination more frequently while individuals with a positive ER style use it to a lesser extent. Our findings are partially consistent with this hypothesis since, as we expected, adolescents’ negative ER style and rumination use in their day-to-day life were positively linked. However, no statistically significant correlation was found between positive ER style and rumination use; although adolescents with a higher predisposition to use positive ER strategies are more likely to engage less in negative strategies (e.g., rumination; [33]), results measured at the “trait” level may be independent of those measured at the “state” or “within-person” level [76,77]. In other words, adolescents with a certain ER style can use both positive and negative ER strategies depending on the situation, and this may be reflected in our findings.

Moreover, we expected that both ER style and rumination use during daily life would be related to temperament. Specifically, we expected NA to be associated with a more negative ER style and greater rumination use while we expected PA to be associated with a greater positive ER style and less rumination use. Our results revealed that adolescents scoring high in NA are more engaged in a dysfunctional style and increased use of rumination, while a higher PA score was associated with greater involvement in a positive ER style and less rumination use, confirming the previous hypotheses. These findings are important because, although previous studies have shown the role of temperament in the preference of ER strategies that children and adolescents initiate to cope with stressful situations (e.g., [49,50]), this is, to our knowledge, the first study to relate temperament to rumination use during adolescents’ daily life.

The second aim of this study sought to investigate how temperament is related to depressive and anxious symptoms, and whether these relationships are mediated by ER style and rumination use under ecological conditions. It was expected that adolescents scoring high in NA would be prone to engage in a negative cognitive ER style, which would lead them to use rumination more often under ecological-negative conditions, consequently increasing the presence of depressive and anxiety symptoms. Conversely, we hypothesized that adolescents scoring high in PA would be prone to engage in a positive cognitive ER style, which would lead them to use less rumination under ecological-negative conditions, resulting in lower depressive symptoms and lower anxiety symptoms.

Considering the inexistence of similar empirical studies, the findings of this work help to elucidate the role of both temperament and ER (examined with both a questionnaire and EMA) in depressive and anxiety symptoms. Theoretically, certain coping strategies (e.g., rumination) are just as likely to lead to emotional problems, as the other way around. The data found revealed that the relationship between NA and depressive symptoms was significantly mediated by a negative ER style and rumination use sequentially, providing strong support for our hypotheses. Regarding rumination, it should be noted that no direct effect of NA on the use of rumination in daily life was observed. The results revealed that a negative ER style mediated the relationship between NA and rumination frequency use. The strong positive association found between NA and negative ER style might suggest that NA intensity determines, to some extent, the degree to which emotions are controlled with a negative ER style [28,39,78,79], which, in turn, determines whether adolescents use rumination in ecological-negative conditions. However, if NA is not high enough to activate the negative ER style by itself it does not explain the involvement of adolescents in the use of rumination in daily life.

Regarding anxiety, with our findings we can conclude that adolescents scoring high in NA report more anxiety symptoms. As discussed above, these results extend the evidence found regarding NA as a risk factor for anxiety symptoms (e.g., [13,18]). However, although previous studies found that the relationship between NA and anxiety symptoms is mediated by a negative ER style (e.g., [28]), our findings revealed a non-significant relationship between those variables. Similarly, as far as rumination is concerned, although McLaughlin et al. [46] previously indicated that ruminative responses to distress predict increased anxiety symptoms, we did not find a direct effect of ruminative use during the adolescents’ day-to-day life on anxiety symptoms. These contradictory findings may be attributable to different methodologies, since the authors mentioned above used only retrospective reports on the relationship between rumination and anxiety symptoms, as opposed to the contextual approach used in this case. Since Tan et al. [54] suggested that rumination use during adolescents’ daily life could be a risk factor for anxiety as well, this issue should be addressed in more depth in future research.

A different pattern emerged from the relationship between PA, ER, and depressive symptoms. As discussed above, a positive ER style was not included as a mediating variable since it showed no significant correlation with rumination use. In this case, both PA and rumination use had significant direct effects on depressive symptoms; furthermore, the relationship between PA and depressive symptoms was mediated by rumination use, indicating that adolescents scoring high in PA used less rumination under ecological-negative conditions, which in turn prompted them to experience lower depressive symptoms. Although previous studies have already shown the negative association between PA and depressive symptoms [13,17] as well as between PA and emotion dysregulation, e.g., use of rumination [29], this is, to our knowledge, the first study to find that PA works as a protective factor for depression since the tendency to experience positive emotions, enthusiasm, and excitement favors the decrease of dysfunctional strategies’ use such as rumination during daily life.

Despite this study’s novel contributions, it does have certain limitations. Firstly, we admit that given the transversal nature of most of our variables, it is difficult to conclude the mediation analyses carried out in terms of causality; it would be interesting to complete this research with other longitudinal studies. Furthermore, our data rely only on self-report measures, which creates the potential for inflated shared-source variance. However, it should be noted that although all the measures selected in this study were reported by adolescents, different methods of obtaining information were used. Measuring the rumination use in adolescents’ lives through an EMA approach allowed to reduce the problem of share-method variance. Although self-reports are considered an appropriate instrument for adolescents [80], it should not be overlooked that response tendencies and social desirability may influence the data, as well as current moods and metacognitive factors [81]. Therefore, it would be interesting to include additional measures such as physiological variables associated with the use of rumination during the momentary ecological study. Moreover, it would be interesting for future research to replicate the present findings using alternative sources of information on adolescent psychopathology (e.g., parent or teacher reports), which would extend the validity and generalizability of these findings. Finally, in our study we focused only on the use of rumination. However, we did not consider other ER strategies highlighted in the literature. For instance, increased use of cognitive reappraisal may be beneficial with respect to symptoms of depression and anxiety in youth (for a review, see [38]). Future research might expand the current findings by examining the role of other ER strategies (e.g., cognitive reappraisal) used during adolescents’ everyday life.

## 5. Conclusions

Despite the above-mentioned constraints, this study has several strengths and it constitutes an attempt to provide further insight into the relationships between temperament (i.e., PA and NA), ER (examined through a questionnaire, as well as during daily life), and depressive and anxious symptoms in adolescence. Our study shows that (1) both a negative ER style and rumination use mediate the relationship between NA and depression; (2) only NA had a significant direct effect on anxiety; (3) PA buffered the effect of rumination use on depression. By evaluating the rumination use in the adolescents’ daily lives, this study provides key information to better understand the patterns of ER in adolescence; patterns which may be targeted by prevention on early interventions efforts, thus averting more severe disorders during adulthood. Moreover, ER interventions would benefit if they may be adapted toward adolescents with certain temperamental traits that may predispose them to psychopathology. Therefore, with a view to improving preventive interventions, the current findings suggest that it is essential to both empower at-risk adolescents (e.g., high NA, low PA, and high negative ER style) in the use of more adaptive strategies to reduce rumination during daily life, as well as to enhance protective factors (e.g., low NA, high PA, and low negative ER style) that buffer the effect of using rumination on the development of depressive and anxious symptomatology.

## Figures and Tables

**Table 1:** Preliminary descriptive statistics.

Measure	*M*	*SD*	Skewness	Kurtosis
NA	16.292	4.591	0.660	−0.471
PA	22.861	3.111	−0.149	−0.206
NERstyle	37.510	10.140	0.269	−0.601
PERstyle	62.970	13.181	0.191	−0.520
RumUse	0.280	0.284	0.908	0.062
DepSymp	6.892	5.199	1.590	2.810
AnxSymp	22.800	16.319	1.660	3.430

NA = negative affect, PA = positive affect, NER style = negative emotion regulation style, PER style = positive emotion regulation style, RumUse = the relative frequency of rumination use, DepSymp = depressive symptoms, AnxSymp = anxiety symptoms.

**Table 2:** Zero-order, pairwise correlations between all measures of interest.

Measure	1	2	3	4	5	6	7
1. NA	−						
2. PA	−0.166	−					
3. NERstyle	0.708 **	−0.006	−				
4. PERstyle	0.132	0.478 **	0.400 **	−			
5. RumUse	0.312 *	−0.284 *	0.444 **	−0.019	−		
6. DepSymp	0.705 **	−0.292 *	0.725 **	0.141	0.527 **	−	
7. AnxSymp	0.726 **	−0.004	0.749 **	0.302 *	0.341 **	0.806 **	−

NA = negative affect, PA = positive affect, NER style = negative emotion regulation style, PER style = positive emotion regulation style, RumUse = the relative frequency of rumination use, DepSymp = depressive symptoms, AnxSymp = anxiety symptoms. * *p* < 0.05, ** *p* < 0.01.

**Table 3:** Results of regression analyses.

Predictor	Coefficient *B*	*SE*	*t*	*p*
Model 1: Depressive symptoms as outcome variable				
NA	0.108	0.109	0.988	0.327
NERstyle	0.858	0.052	1.624	0.109
RumUse	3.123	1.293	2.414	0.018
Model 2: Anxiety symptoms as outcome variable				
NA	0.770	0.370	2.082	0.041
NERstyle	0.319	0.193	1.646	0.105
RumUse	−5.719	4.609	−1.240	0.219
Model 3: Depressive symptoms as outcome variable				
PA	−0.374	0.109	−3.423	0.001
RumUse	3.123	1.293	2.414	0.018

NA = negative affect, PA = positive affect, NER style = negative emotion regulation style, RumUse = the relative frequency of rumination use.

**Table 4:** Bootstrapped indirect effects of adolescent affect on depressive and anxious symptoms through mediators.

Mediator	Types of Affect	Bootstrapping Percentile 95 CI	
		Upper	Lower
Models 1 and 3: Depressive symptoms as outcome variable			
NERstyle	NA	−0.019	0.190
RumUse	NA	−0.106	0.054
	PA	−0.192	−0.009
NERstyle-RumUse	NA	0.002	0.089
Models 2: Anxious symptoms as outcome variable			
NERstyle	NA	−0.068	0.696
RumUse	NA	−0.106	0.316
NERstyle-RumUse	NA	−0.190	0.043

NA = negative affect, PA = positive affect, NER style = negative emotion regulation style, RumUse = the relative frequency of rumination use.
